# Characterization of Transcriptional Repressor Gene *MSX1* Variations for Possible Associations with Congenital Heart Diseases

**DOI:** 10.1371/journal.pone.0142666

**Published:** 2015-11-10

**Authors:** Fei-Feng Li, Ying Han, Shuai Shi, Xia Li, Xi-Dong Zhu, Jing Zhou, Qing-Liang Shao, Xue-Qi Li, Shu-Lin Liu

**Affiliations:** 1 Genomics Research Center (one of the State-Province Key Laboratory of Biopharmaceutical Engineering, China), Harbin Medical University, Harbin, China; 2 Translational Medicine Research and Cooperation Center of Northern China, Heilongjiang Academy of Medical Sciences, Heilongjiang, China; 3 Department of Cardiology, the Fourth Affiliated Hospital of Harbin Medical University, Harbin, China; 4 Department of Neurology, the Second Affiliated Hospital of Harbin Medical University, Harbin, China; 5 Intensive care unit, the Second Affiliated Hospital of Harbin Medical University, Harbin, China; 6 Department of Neonatalogy, the Second Affiliated Hospital of Harbin Medical University, Harbin, China; 7 Department of Microbiology, Immunology and Infectious Diseases, University of Calgary, Calgary, Canada; University of Texas at Austin Dell Medical School, UNITED STATES

## Abstract

**Background:**

The human heart consists of several cell types with distinct lineage origins. Interactions between these cardiac progenitors are very important for heart formation. The muscle segment homeobox gene family plays a key role in the cell morphogenesis and growth, controlled cellular proliferation, differentiation, and apoptosis, but the relationships between the genetic abnormalities and CHD phenotypes still remain largely unknown. The aim of this work was to evaluate variations in *MSX1* and *MSX2* for their possible associations with CHD.

**Methods:**

We sequenced the *MSX1* and *MSX2* genes for 300 Chinese Han CHD patients and 400 normal controls and identified the variations. The statistical analyses were conducted using Chi-Square Tests as implemented in SPSS (version 19.0). The Hardy-Weinberg equilibrium test of the population was carried out using the online software OEGE.

**Results:**

Six variations rs4647952, rs2048152, rs4242182, rs61739543, rs111542301 and rs3087539 were identified in the *MSX2* gene, but the genetic heterozygosity of those SNPs was very low. In contrast, the genetic heterozygosity of two variations rs3821949 near the 5’UTR and rs12532 within 3’UTR of the *MSX1* gene was considerably high. Statistical analyses showed that rs3821949 and rs12532 were associated with the risk of CHD (specifically VSD).

**Conclusions:**

The SNPs rs3821949 and rs12532 in the *MSX1* gene were associated with CHD in Chinese Han populations.

## Introduction

Congenital heart diseases (CHD) are a group of complex congenital anatomic malformations worldwide with high morbidity and mortality. The incidence of the illness is about 7.5% in newborns [1] and 1% of the patients required clinical intervention [2]. There are numerous types of this disease, including ventriculap septal defect, pulmonary stenosis, tetralogy of Fallot, patent ductus arteriosus, mitral valve insufficiency, etc. [3], which are often complicated with arrhythmias and heart failure [4]. So far, many gene mutations and chromosomal variants have been identified in familiar and sporadic CHD cases [5–7]. However, the relationships between those genetic abnormalities and CHD phenotypes still remain largely unknown.

The mammalian heart is a complex and also one of the first formed organs during embryogenesis [3], and the formation is strictly regulated and controlled by gene regulatory networks, consisting of signaling pathways, transcription factors, epigenetic factors and miRNAs [8, 9]. Among the regulatory networks, the Nodal/TGF-βsignaling pathway has a key role in early stages of human embryonic stem (HES) cell differentiation, directing the cells to develop into different embryonic lineages. Any malfunctions in the pathway may lead to errors in the transformation of the embryonic lineages [10–12]. For example, defects in the transforming growth factors LEFTY in the Nodal/TGF-β signaling pathway may affect the signaling of NODAL and TGF-β[3, 13]. In a previous study, we found that single nucleotide polymorphisms (SNPs) of the *Lefty* genes are associated with the risk of CHD [3]. The *Nodal* gene in the Nodal/TGF-β signaling pathway can initiate a series of signal transduction events in the later stages of embryonic development [13, 14]. However, no variations in the *Nodal* gene so far have been associated with the risk of CHD [3]. Therefore, it is not clear whether it is the Nodal/TGF-β signaling pathway or only *Lefty* that is associated with the risk of CHD. Additionally, SMAD3, an intracellular regulating factor in the Nodal/TGF-β signaling pathway to modulate the transcription of many genes [15, 16], together with LEFTY plays central roles in the signaling pathway [15, 17]. Our previous work has demonstrated that the variant rs2289263 before 5’UTR of the *SMAD3* gene is associated with increased risk of VSD in the Chinese Han population [18]. Additionally, as the process of HES cell differentiation during embryonic development is very important for the heart development, it may also be involved in the pathogenesis of CHDs.

During embryonic development, HES cells differentiate to various cell types of ectoderm, endoderm and mesoderm, and the cardiomyocytes are generated and differentiated in the mesoderm [19]. As the heart consists of several cell types with distinct lineage origins [20], such as myocardium cells, cardiac neural crest (NC) cells, aorticopulmonary septum cells and membranous ventricular septum cells, etc. [21], interactions between these cardiac progenitors are very important for the cardiac development, and any mistakes may result in congenital heart malformations [1]. The muscle segment homeobox gene family is an important transcriptional regulator during embryonic development and has an important role in cell morphogenesis and growth [22]. Muscle segment homeobox 1 (MSX1) and Muscle segment homeobox 2 (MSX2) are members of the muscle segment homeobox gene family that encode transcription factors, playing important roles in the organogenesis and tissue–tissue interactions during vertebrate embryonic development [23], and mutations in *MSX1* or *MSX2* have been associated with impaired development of cranial neural crest-derived structures, oral clefts, and nonsyndromic oligodontia [23–27]. Studies with animal models also identified *MSX1* and *MSX2* double mutants in a broad range of heart malformations, such as tetralogy of Fallot and persistent truncus arteriosus [20, 23].

In this study, we analyzed the transcribed regions and splicing sites of the *MSX1* and *MSX2* genes and compared the sequences between 300 Chinese Han CHD patients and 400 controls to validate the possible associations of *MSX1* and *MSX2* with CHDs,. We found that variations rs3821949 near the 5’UTR and rs12532 within the 3’UTR of the *MSX1* gene were closely associated with the risk of CHD (specifically, VSD).

## Results

### Patients

Clinical diagnosis of all recruited members was confirmed at the Fourth or the Second Affiliated Hospitals of Harbin Medical University. The CHD patients had no history or manifestations of any other systemic abnormalities. We also established that their mothers did not take medicines or attract infections during gestation, because these factors had been found to be associated with heart malformation in pregnancy [28, 29].

A total of 300 CHD patients (male 136, female 164, the min and max age were 0.2 and 61.0 respectively, and the average age was 15.47 years) and 400 unrelated controls (male 173, female 227, the min and max age were 0.3 and 60.0 respectively, and the average age was 13.68 years) were recruited for this study, and there was no statistical differences of the gender composition and age between the two groups (Table 1). The 300 CHD patients contained 128 with ventricular septal defects (VSD), 107 with atrial septal defects (ASD), 44 with patent ductus arteriosus (PDA), 6 with tetralogy of Fallot, 4 with pulmonary stenosis, and 11 with other types of congenital heart defects.

**Table 1:** Clinical characteristics and analysis of the study population.

*Parameter*	*CHD*	*Control*	*F*	*t*	*P*	*95%CI-Up*	*95%CI-Low*
***Sample (n)***	300	400	-	-	-	-	-
***Male/Female (n)***	136/164	173/227	-	-	0.583	-	-
**Age (years)**	15.47±17.47	13.68±10.22	147.419	-1.579	0.115	-4.00676	0.43695

Data are shown as mean±SD; between the two groups, there were no statistical differences of the age and gender composition.

### *MSX1* and *MSX2* gene analysis

**Fig 1:**
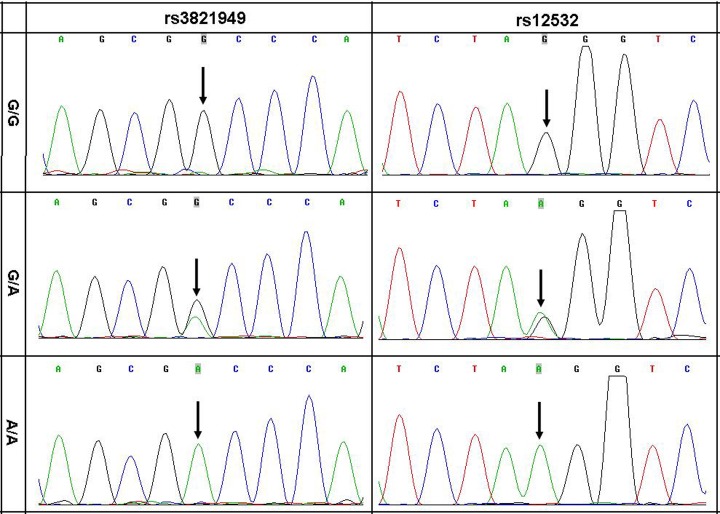
Three genotypes of DNA sequence chromatograms of rs3821949 and rs12532.

We sequenced the *MSX1* and *MSX2* genes to test the hypothesis that germline common genetic variants in *MSX1* or *MSX2* may confer susceptibility to CHD. Upon analyzing the transcribed regions and splicing sites of *MSX1* and *MSX2*, we identified rs4242182, rs61739543 and rs111542301 within the translated region, and rs2048152 within an intron, and rs4647952 and rs3087539 within 5’UTR and 3’UTR respectively of the *MSX2* gene, but the genetic heterozygosity of all these SNPs loci was low. On the other hand, we identified rs3821949 near 5’UTR and rs12532 within 3’UTR of the *MSX1* gene, and the genetic heterozygosity of the two SNPs was considerably high (Fig 1).

### SNP rs3821949 and rs12532 genotyping statistical analysis

To test possible associations between *MSX1* and CHD, we conducted SNP analyses and found that both rs3821949 and rs12532 were associated with the risk of CHD (specifically VSD) in Chinese Han population (Tables 2 and 3).The Hardy-Weinberg equilibrium test for the CHD and controls were conducted and it was in line with the equilibrium.

**Table 2:** The genotype and allele frequency of SNP rs3821949 and rs12532 in 300 Chinese Han CHD patients and 400 non-CHD controls.

*SNP*	*Group*		*Genotype frequency (%)*			*Allele frequency (%)*	
***rs3821949***	Genotype		G/G	G/A	A/A	G	A
	CHD	300	189(63.0)	74(24.7)	37(12.3)	452(75.3)	148(24.7)
	VSD	128	81(63.3)	31(24.2)	16(12.5)	193(75.4)	63(24.6)
	ASD	107	63(58.9)	29(27.1)	15(14.0)	155(72.4)	59(27.6)
	Controls	400	292(73.0)	72(18.0)	36(9.0)	656(82.0)	144(18.0)
***rs12532***	Genotype		G/G	G/A	A/A	G	A
	CHD	300	121(40.3)	139(46.3)	40(13.3)	381(63.5)	219(36.5)
	VSD	128	55(43.0)	62(48.4)	11(8.6)	172(67.2)	84(32.8)
	ASD	107	31(29.0)	64(59.8)	12(11.2)	126(58.9)	88(41.1)
	Controls	400	126(31.5)	219(54.8)	55(13.8)	471(58.9)	329(41.1)

**Table 3:** SNP rs3821949 and rs12532 within MSX1 gene associated with the risk of congenital heart diseases in Chinese populations.

*Title*			*Pearson Chi-square*				*Pearson’s R*			
*Genotyped SNP*	*Disease Type*	*Statistical Types*	*Value*	*Min count* ^a^	*df*	*Asymp*. *Sig*. *(2-sided)*	*Value*	*Asymp*. *Std*. *error* ^b^	*Approx*. *T* ^c^	*Approx*. *Sig*
***rs3821949***	CHD-Control	Genotype	7.974	31.29	2	0.019	-0.098	0.038	-2.606	0.009^d^
		Allele	9.231	125.14	1	0.002	-0.081	0.027	-3.046	0.002^d^
	VSD-Control	Genotype	4.425	12.61	2	0.109	-0.086	0.045	-1.976	0.049^d^
		Allele	5.376	50.18	1	0.020	-0.071	0.032	-2.322	0.020^d^
	ASD-Control	Genotype	8.029	10.76	2	0.018	-0.118	0.047	-2.661	0.008^d^
		Allele	9.657	42.84	1	0.002	-0.098	0.034	-3.119	0.002^d^
***rs12532***	CHD-Control	Genotype	6.187	40.71	2	0.045	0.069	0.038	1.825	0.068^d^
		Allele	3.079	234.86	1	0.079	0.047	0.027	1.755	0.079^d^
	VSD-Control	Genotype	6.509	16.00	2	0.039	0.110	0.042	2.536	0.012^d^
		Allele	5.627	100.12	1	0.018	0.073	0.030	2.376	0.018^d^
	ASD-Control	Genotype	0.972	14.14	2	0.615	0.000	0.043	0.001	0.999^d^
		Allele	0.000	88.01	1	0.999	0.000	0.031	0.001	0.999^d^

a: The minimum expected count

b: Not assuming the null hypothesis

c: Using the asymptotic standard error assuming the null hypothesis

d: Based on normal approximation

## Discussion

In this study, we analyzed the transcribed regions and splicing sites of the *MSX1* and *MSX2* genes in a large cohort of CHD patients and controls, and found that the variations rs3821949 and rs12532 in the *MSX1* gene were associated with the risk of CHD in the Chinese Han population, demonstrating the involvement of the *MSX1* gene in the CHD etiology.

The mammalian heart is a complex organ, starting to form in the mesoderm 18 or 19 days after fertilization, and many genes with strict temporal, spatial, and sequential expression are involved in the formation [3]. The genes *MSX1* and *MSX2* encode the transcription factors that play key roles in the survival and differentiation of secondary heart field precursors [20, 30]. *MSX1* and *MSX2* double mutants in mice show malposed, elongated or spiral rotated heart outflow tract [20, 31, 32], and *MSX1* and *MSX2* null mutants in embryos can increase apoptosis in the secondary heart field, hence leading to many heart malformations [33]. Mutations in *MSX1* and *MSX2* may perturb the differentiation of heart field precursors and myocardial cells in the heart outflow tract [34–36], while mice with mutation in only *MSX1* or *MSX2* did not show obvious cardiac defects [24, 25], suggesting that there may be some complementary roles of the MSX1 and MSX2 transcription factors in heart development. However, in this study, the genetic heterozygosity of the SNPs located within the *MSX2* gene was very low in the Chinese Han population.

LEFTY and SMAD3 play important roles in the Nodal/TGF-Lefty signaling pathway [3, 13, 15–18] and we have previously found that SNPs in *Lefty* and *SMAD3* genes are associated with the risk of CHD [3, 18]. Normal functions of the Nodal/TGF-β signaling pathway are very important for the early stages of HES cell differentiation into different embryonic lineages [10, 12]. During embryonic development the HES cells differentiate to various cell types including cardiomyocytes in the mesoderm [19]. The heart consists of several cell types with distinct lineage origins [20, 21], and interactions between these cells are very important for the cardiac development [1]. The muscle segment homeobox gene family controls cell morphogenesis, growth, proliferation, differentiation, and apoptosis during embryonic development [22]. So the differentiation of HES cells and cardiac progenitor cell interactions during embryonic development are important for the heart development and any defects or mistakes may cause CHDs.

*MSX1* seems to be an active gene in the HES cells as implicated by its up-regulated expression when the HES cells are co-cultured with PA6 cells [37], and, conversely, the expression levels of MSX1 become lower when the HES cells are treated with dopamine [38]. In a genome-wide methylation-gene expression study between the epigenetic modifications of retinoic acid treated and undifferentiated HES cells, the author uncovered 166 differentially methylated CpG sites and 2,013 differentially expressed genes, in which 19 genes including MSX1 are highly correlated with each other [39].

The 3’UTR and 5’UTR sequences play important roles in regulating the expression of genes [40] [41]. Of great significance, variations within 3’UTR may be associated with human tumorigenesis and survival of the patient [42, 43]. The 5′UTR region of gene is a structural complex, which can bind with some miRNAs and may be involved in gene expression, protein translation and disease pathogenesis [44, 45]. In a recently study, we demonstrated that the variant rs2289263 before the 5’UTR of *SMAD3* gene is associated with increased risk of VSD in the Chinese Han population[18]. Probably, the SNPs in 3’UTR or 5′UTR may affect the binding of the untranslated regions with regulatory factors such as miRNAs and eventually hamper the gene expression and function. Findings in this study with rs3821949 and rs12532 of the *MSX1* gene have updated our understanding on 5’UTR and r 3’UTR and may lead to new insights into the pathogenesis of CHDs.

## Materials and Methods

### The study population

From the Fourth and the Second Affiliated Hospitals of Harbin Medical University, Harbin, China, we collected specimens of 300 CHD patients and 400 normal controls for this study (Table 1); specimens of 90 of the 300 CHD patients were overlapping with those used for another study [3, 18]. All the CHD patients and normal controls were given comprehensive physical examination, electrocardiogram and ultrasonic echocardiogram examinations. None of the patients showed any other cardiac or systematic abnormalities, and the normal controls did not show any defects in the heart or other body parts. For this work, we obtained a written informed consent from each participant or their parents on behalf of minors, and the Ethics Committee of the Harbin Medical University approved this work, consistent with the 1975 Declaration of Helsinki.

### DNA analysis

Using standard protocols, we extracted the genomic DNA from the peripheral blood leukocytes of the participants. The human *MSX1 and MSX2* genes each consist of two exons located on 6p16.2 and 5q35.2, respectively. To determine the SNP genotypes in the genes, we amplified the four exons and splicing sites of the genes using polymerase chain reaction (PCR) method, and sequenced the products using standard protocols [46]. After that, the genotypes of the SNP were determined using PCR and gene sequencing methods [3].

### Rs3821949 and rs12532 SNP genotyping analysis and Statistical methods

**Fig 2:**
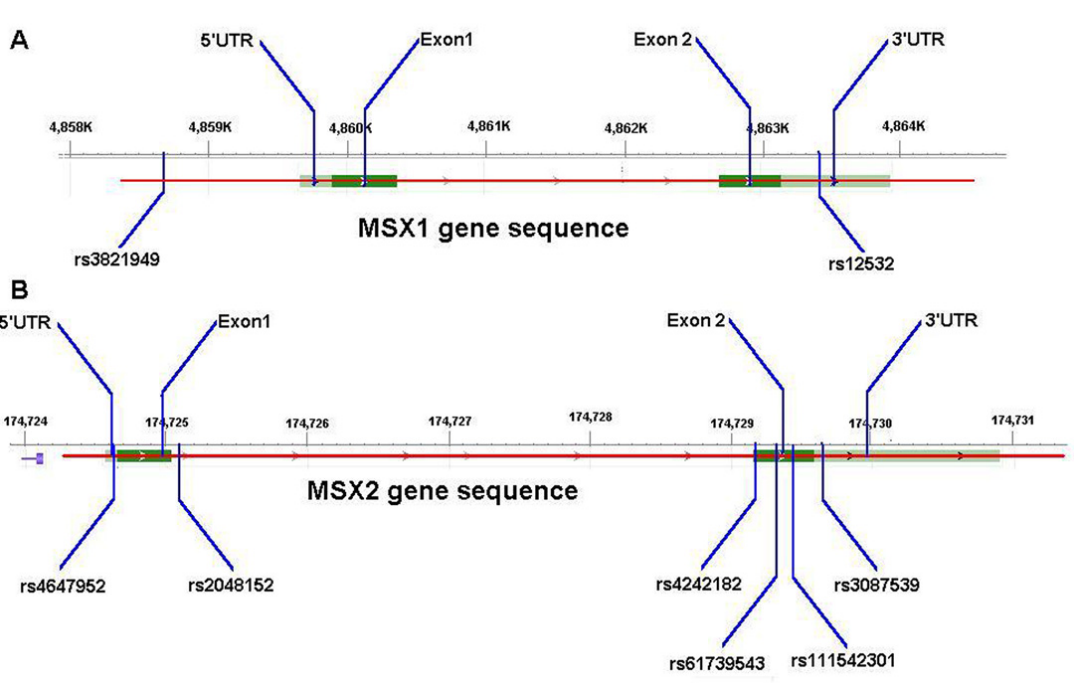
Schematic diagrams of SNP in the genes. A: locations of rs3821949 and rs12532 in the MSX1 gene; B: locations of rs4647952, rs2048152, rs4242182, rs61739543, rs111542301 and rs3087539 in the MSX2 gene.

We determined genotypes of the rs3821949, rs12532 and rs4647952, rs2048152, rs4242182, rs61739543, rs111542301, rs3087539 in the *MSX1* and *MSX2* genes (Fig 2), and all the measurements were conducted by two independent researchers (Table 4). And then overall CHD meta-analysis was conducted according to the types of CHD and sample sizes.

**Table 4:** PCR primers used for SNP statistical analysis.

*Gene*	*SNPs*	*Directions*	*primers*	*Size*	*Tm (°C)*
***MSX1***	Rs3821949	*Forward*	CTCCCCTGACCCCAACTC	388bp	52.8
		*Reverse*	CCACCCTCGCTCTGAACT		
	Rs12532	*Forward*	CTCCGAAGTCTGATCCCT	215 bp	51.0
		*Reverse*	CTTTTCTTGCCTGGTGT		
***MSX2***	Rs4647952	*Forward*	TTCGGGAAGAGCCAATCA	376 bp	59.4
		*Reverse*	TTCTTGTCGGACATGAGCG		
	Rs2048152	*Forward*	CAGAGGGTGTCTTGGATGCG	249 bp	59.3
		*Reverse*	AGATGGAGCGGCGTGGAT		
	Rs4242182	*Forward*	AGAGGGGAGGCCCGAAAG	203 bp	53.9
		*Reverse*	GCGAGGAGCTGGGATGTG		
	Rs61739543	*Forward*	ACGCCCTTTACCACATCC	542 bp	55.3
		*Reverse*	CCTCCGCCTACAGAACAA		
	Rs111542301	*Forward*	ACCACATCCCAGCTCCTC	346 bp	56.8
		*Reverse*	GGTACATGCCATATCCCACT		
	Rs3087539	*Forward*	GCATGTACCACCTGTCCT	268 bp	52.5
		*Reverse*	CCATCAGAGCCAATCTTT		

The continuous variable (measurement data, such as age) statistical analyses were conducted using independent-samples T test and the discrete variable (enumeration data, such as gender composition and genotype frequency) statistical analyses were conducted using Chi-Square Tests to calculate odds ratios and P value as implemented in SPSS (version 19.0). P values less than 0.05 were considered statistically significant. The Hardy-Weinberg equilibrium test of the CHD and control population was conducted with the online software OEGE.
